# The nature of ‘jaws’: a new predatory representative of Provora and the ultrastructure of nibbling protists

**DOI:** 10.1098/rsob.240158

**Published:** 2024-12-18

**Authors:** Artem O. Belyaev, Sergey A. Karpov, Patrick J. Keeling, Denis V. Tikhonenkov

**Affiliations:** ^1^ Papanin Institute for Biology of Inland Waters, Russian Academy of Sciences, Borok, Yaroslavl, Russia; ^2^ Zoological Institute, Russian Academy of Sciences, Saint Petersburg, Russia; ^3^ Department of Botany, University of British Columbia, Vancouver BC V6T 1Z4, Canada

**Keywords:** ultrastructure, evolution of eukaryotes, Nibbleridia, cytoskeleton, microtubular roots, 18S rRNA phylogeny

## Abstract

The recently discovered Provora supergroup has primarily been examined to determine their phylogenomic position in the eukaryotic tree. Their morphology is more poorly studied, and here we focus on their cellular organization and how it compares with that of other supergroups. These small eukaryovorous flagellates exhibit several ultrastructural features that are also found in a subset of taxa from a wide variety of deep-branching lineages (Stramenopiles, Alveolata, Hemimastigophora, Malawimonadidae, Discoba and Metamonada), including vesicles beneath the plasmalemma, two opposing vanes on the flagella, a ventral feeding groove and a fibrillar system resembling the excavate type. Additionally, we identified four main microtubular roots (r1–r4) and a singlet root between r1 and r2, which support the strong feeding apparatus resembling ‘jaws’. Their unique extrusive organelles (ampulosomes) have a similar organization to Hemimastigophora extrusomes, but most of their cell characteristics most closely resemble features of the TSAR + Haptista grouping. We also describe a new species, *Nibbleromonas piranha* sp. nov., and highlight features of its feeding behaviour, which can be so aggressive as to result in cannibalism.

## 1. Introduction

The search for new lineages of eukaryotes and reconstruction of their cell ultrastructure are extremely important for understanding phylogenetic relationships and how the diversity of cellular forms evolved. The investigation of the flagellar apparatus is of particular importance since it has a highly conserved core, but also exhibits a great diversity of evolutionarily important associated structures, such as the system of microtubular roots and fibrils [1]. Small changes in conserved structures of the flagellar apparatus often reflect the divergence of major evolutionary lineages [2]. In addition, morphological and ultrastructural adaptations to various ecological and physical conditions are associated with behavioural features, including different strategies for nutrition and life cycles [3–5].

Provora is a newly discovered supergroup of small unicellular eukaryotes consisting of two genetically and morphologically distinct subgroups: Nebulidia and Nibbleridia. Currently, only seven species of Provora have been described, but according to environmental sequence data, there are dozens of unknown species, genera and families living almost everywhere in marine environments, and their structure and behaviours remain to be elucidated [6,7]. Provorans are small, fast-swimming and superficially unremarkable rounded biflagellates. These flagellates are generally found at low abundance but may still play an environmentally significant role in aquatic ecosystems worldwide because they consume bacteriotrophic protists and serve as energy transmitters to higher levels of the microbial loop [6,8]. Remarkably, small eukaryovorous nibblerids can ingest prey larger than themselves. They possess an unusual type of phagotrophic nutrition characterized by the biting of a portion of the prey cell, mediated by a robust cytostome apparatus (‘jaws’), which has attracted considerable interest in the study of the cytoskeleton system of these organisms. In protists, there are three widely recognized modes of food capture: phagocytosis, which is observed in most predators; myzocytosis, which is observed in some alveolates and rhizarians; and trogocytosis, which is exhibited by a few heterolobosean amoebas [9–11]. The latter is most similar to the biting behaviour observed in Nibbleridia but is fundamentally different in its mechanism since nibblerids use the ventral groove to bite off most of the cell, including the cytoplasmic content.

The ventral feeding groove and flagellar folds of nibblerids (and nebulids) resemble those found in excavates and eukaryovorous colponemids and are considered to be among the earliest traits of the first eukaryotes [12–16]. It appears that the vanes (folds) increase the efficiency of the flagellum in creating a water current to pull bacteria into the feeding groove [14]. Despite their similar feeding-associated structures, Provora, colponemids and excavates are very distantly related. Similarly, the provorans also contain filamentous inclusions in their mitochondrial cristae that are also known in some Stramenopiles [17,18], which are also only very distantly related. Overall, the ultrastructural features of the provorans probably include a number of ancient morphological traits that are differentially retained in several major branches of the eukaryotic tree [6].

Given the limited research on the diversity of Provora, characterizing new representatives of this novel supergroup is also of critical importance. Given the depth of this branch of the eukaryotic tree, Provora might be expected to differ significantly from each other, as do members of other supergroups like stramenopiles and alveolates. Additionally, expanding the taxonomic sampling of Provora is important for addressing the challenging problem of their phylogenetic relationships, which currently suggest some relationship to TSAR, Haptista and Hemimastigophora, but the exact nature of this is unresolved. Here we report a new species of Nibbleridia, *Nibbleromonas piranha* sp. n. from the marine waters of Korea and present a detailed study of the ultrastructure of the genus *Nibbleromonas* spp. We also show that these flagellates possess aggressive feeding resulting in cannibalism.

## 2. Methods

### 2.1. Establishing clonal cultures

Clonal culture of *Nibbleromonas piranha* sp. n. (strain Jim-2) was isolated from a sample of coastal marine sediments of the Sea of Japan, Jeodo Island, Republic of Korea, with a salinity of 22‰ on 10 May 2019 (35°03′09.86″N, 128°33′47.24″E) and established using *Procrybtobia sorokini* (Zhukov 1975) Frolov et al. 2001 as prey, as described previously [6]. The sample was collected within the framework of the Russian–Korean bilateral cooperation Basic Science Research Program through the National Research Foundation of Korea (NRF) and Russian Foundation for Basic Research.

All studied strains of nibblerids, including Jim-2 and those obtained earlier [6], were cultivated under the same conditions (temperature of 22°C, darkness) in marine Schmalz-Pratt medium (pH 7.2; 22‰) with the same prey concentration. Strains are currently stored in a collection of Live Protozoan Cultures at the Papanin Institute for Biology of Inland Waters, Russian Academy of Sciences, and in the University of British Columbia.

### 2.2. Light microscopy and video

Cells were observed using a Zeiss Axioscope A1 and a 63× water immersion objective with phase contrast or DIC. The images and videos were taken with an MC-20 camera (Lomo-Microsystems, Russia) and an MC-1009/S video camera (AVT Horn, Aalen, Germany).

### 2.3. Scanning electron microscopy

Cells were centrifuged and fixed with 2.5% glutaraldehyde in marine Schmalz-Pratt medium (pH 7.2) for 30 min at 22°C. Then, the cells were drawn onto a polycarbonate filter (0.8 μm pores). After that, the cells were dehydrated in an ethanol series (30%, 50%, 70%, 96% and 100%) followed by ethanol with propylene oxide (1 to 1 ratio) for 10 min each and 100% propylene oxide (three times for 10 min each). Cells were then incubated overnight in 100% hexamethyldisiloxane and dried. The dry filters were mounted on aluminium stubs, coated with gold and observed with a JSM-6510LV (JEOL, Tokyo, Japan) electron microscope.

### 2.4. Transmission electron microscopy

For investigation of the ultrathin series, cells were centrifuged for 20 min at 5000×g; 0.5 ml of 4% glutaraldehyde (in 0.1 M cacodylate buffer) was added to 0.5 ml of the resuspended cells and incubated at +4°С for 2 h. The pellet of fixed cells was subsequently embedded in 1% agarose and rinsed twice (10 min each) with cold (4°С) 0.1 M cacodylate buffer. After that, cells were fixed in cold (+4°С) 1% osmium tetroxide in 0.1 M cacodylate buffer for 1 h. Then, the pellet was rinsed with 0.1 M cacodylate buffer for 10 min. After dehydration in an alcohol series (30%, 50%, 70%, 96% and 100%) and propylene oxide, the pellet was embedded in Spurr resin (EM 0300 Sigma-Aldrich). Ultrathin sections (60 nm) were prepared with a Leica EMUC6 ultramicrotome (Leica Microsystems, Germany) and observed using a JEM-1011 (JEOL, Japan).

To observe whole-mount preparations, a drop of cell culture was placed on Formvar-coated transmission electron microscopy grids and fixed in vapours of 2% osmium tetroxide for 10 min. After rinsing with distilled water, cells were stained in 1% uranyl acetate (С_4_H_6_O_6_U) for 20 min and rinsed with distilled water again. Whole-mount preparations were observed by using a JEM-1011 (JEOL, Japan).

### 2.5. 18S rRNA gene sequencing

Cells were harvested from Petri dishes following peak abundance after consuming most of the prey. The cells were collected by centrifugation (1000×g, room temperature) onto the 0.8 µm membrane of a Vivaclear minicolumn (Sartorius Stedim Biotech Gmng, cat. no. VK01P042). Genomic DNA was isolated using the Master Pure Complete DNA and RNA Purification Kit (Epicentre, cat. no. MC85200). The 18S rRNA genes were amplified using the EconoTaq PLUS GREEN 2X Master Mix (Lucigen, cat. no. 30033-1) and universal eukaryotic primers EukA–EukB [19]. Amplified DNA fragments were purified with a QIAquick PCR Purification Kit (Qiagen, cat. no. 433160764). The PCR product was sequenced directly via Sanger dideoxy sequencing. Two additional internal primers, 18SintF (5′-GGTAATTCCAGCTCCAATAGCGTA-3′) and 18SintR (5′-GTTTCAGCCTTGCGACCATACT-3′), were used. The resulting sequences were assembled from four overlapping reads using the Geneious R7 7.0.6 program (https://www.geneious.com).

### 2.6. Phylogenetic analysis

A previously published dataset of all available provorans and some representative eukaryotic sequences was used for phylogenetic reconstructions [6]. Multiple sequence alignment was performed using the L-INS-i algorithm in MAFFT version v. 7.490 [20], and the sequences were trimmed using an automated trimming heuristic followed by a gap threshold filter of 0.7 in TrimAl version 1.4 [21]. Phylogenetic trees were reconstructed using Bayesian and maximum likelihood (ML) methods. Bayesian analysis was performed in MrBayes v. 5.1.16 [22] using the GTR+GAMMA4+I model to calculate posterior probability. Four independent Metropolis-coupled Markov chains were run for 20 million generations and summarized with a 50% burn-in. ML phylogeny was inferred using IQ-TREE (v. 1.6.12) [23,24] and RAxML-NG (v. 1.0.0) [25]. ML reconstruction with IQ-TREE was performed with 1000 ultrafast bootstrap replicates (phylogeny of provorans and eukaryotes) or nonparametric bootstraps with 1000 replicates (phylogeny of provorans) under the TN+F+R3 model, determined by the in-built ModelFinder. ML reconstruction with RAxML-NG was performed with 20 random starting trees for the best ML tree search based on the GTR+GAMMA4+I model.

## 3. Results

### 3.1. Ultrastructure of *Nibbleromonas* spp.

**Figure 1.**
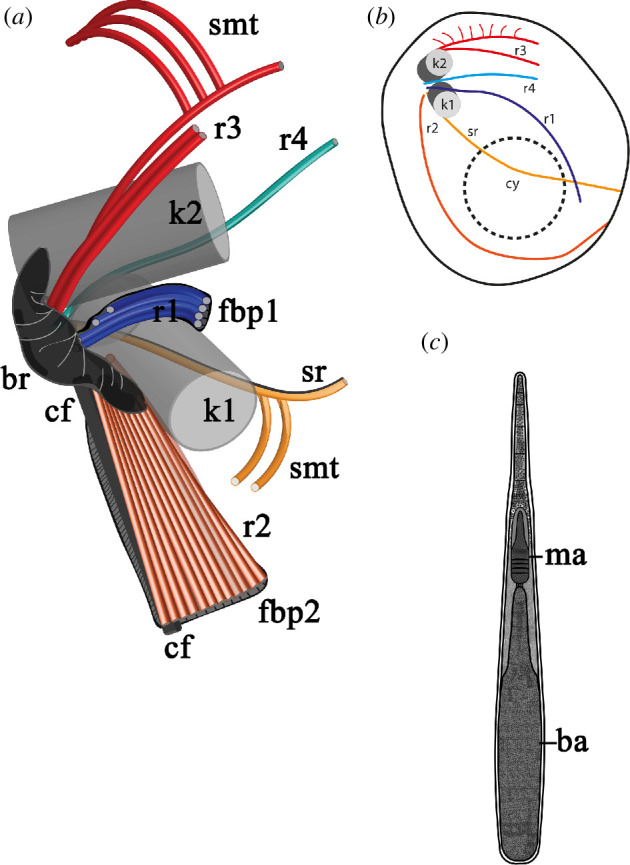
Schematics of microtubular and extrusome organization of *Nibbleromonas* spp. (*a*) Three-dimensional reconstruction of the flagellar apparatus of *Nibbleromonas*. (*b*) General view of the root organization of cell. (*c*) Structure of the ampulosome. Abbreviations: ba: basal ampule of the ampulosome; br: right cross-striated fibrillary bridge between kinetosomes; cf: cytostomal fibre; cy: cytostome; fbp1: fibril plate of root 1; fbp2: fibril plate of root 2; k1: kinetosome of posterior flagellum; k2: kinetosome of anterior flagellum; ma: middle ampule of the ampulosome; r1–r4: roots of flagella; sr: singlet root; smt: secondary microtubules.

All known species of the genus *Nibbleromonas* had similar ultrastructures according to our observations. Most of the data used for the reconstruction of the *Nibbleromonas* spp. cytoskeleton are from *N. quarantinus*, whose transmission electron microscopy preparations were characterized by better fixation quality. We also compared data across different strains to create a more comprehensive model (figure 1*a*,*b*).

### 3.2. Main architecture of the cell

**Figure 2.**
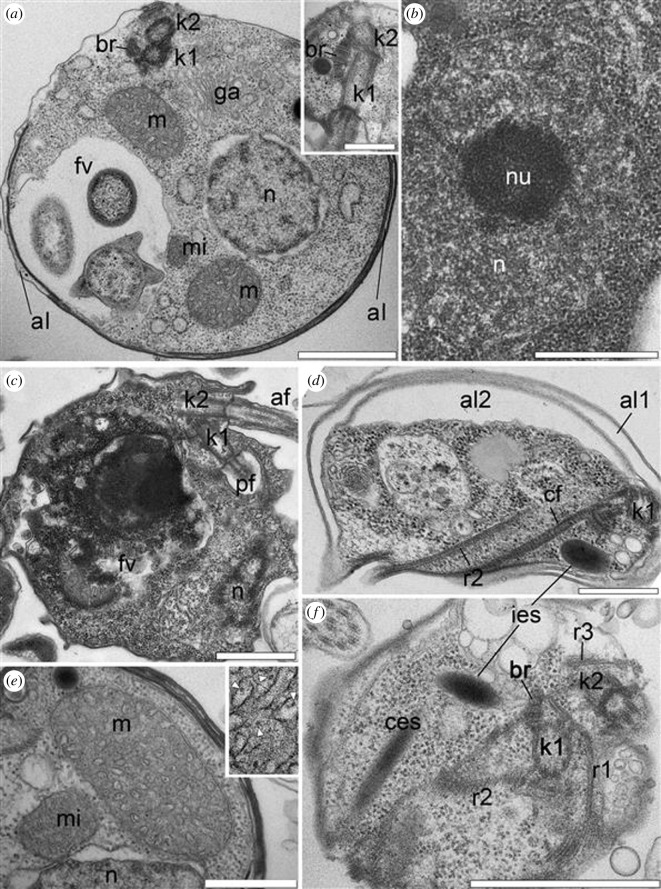
Ultrastructure of the nibblerids *Nibbleromonas quarantinus* (*a,b,c,e,f*) and *N. arcticus* (*d*). (*a*) General disposition of the nucleus, organelles and kinetosomes. The inset shows the structure of the right bridge between the kinetosomes. (*b*) Nucleus and nucleolus structure. (*c*) Dorso-ventral section of the cell with a large food vacuole (fv) containing eukaryotic prey. (*d*) Two layers of alveoli (al1 and al2) and cytostomal structures (cf and r2). (*e*) The structure of the mitochondrion (m) with the filament inside the cristae (arrows) and microbody (mi). The inset shows the filaments inside the cristae (arrows). (*f*) Section through the ventral plane showing the cytostomal cytoskeleton and disposition of two types of extrusomes. Scale bars: (*a*,*c*,*f*) 1 µm; (*a*) (inset), (*b*,*d*,*e*) 0.5 µm. Abbreviations: af: anterior flagellum; al: alveoli embedded within the surface on the dorsal side of the cell; al1: alveoli underlying plasma membrane (external layer), al2: alveoli underlying al1 (inner layer); br: right cross-striated fibrillary bridge between kinetosomes; cf: cytostomal fibre; cm: central microtubules of flagellum; ces: cytostomal extrusomes; ies: interflagellar extrusomes; fv: food vacuole; ga: Golgi apparatus; k1: kinetosome of posterior flagellum; k2: kinetosome of anterior flagellum; m: mitochondrion; mi: microbody; n: nucleus; nu: nucleolus; pf: posterior flagellum; r1–r3: roots of flagella.

The cell surface of *Nibbleromonas* species consists of a plasmalemma underlined by one to three layers of flattened vesicles. These vesicles are primarily found on the dorsal and lateral sides of the cell, while the ventral and subapical parts are usually covered by the plasmalemma only (figure 2*a,d*). In relation to the condition of the cell, the number and size of alveoli vary essentially. They often flatten and produce three to seven membrane layers (including the plasma membrane) covering the cell (figure 2*a,d*). The inner layers of such multimembrane coverings are formed as a result of vesicular transport from the Golgi apparatus and probably promote the formation of food vacuoles during feeding. A dictyosome is located between the flagellar basal bodies (kinetosomes) and the nucleus (figure 2*a*; electronic supplementary material, figure S4*i*). A microbody was adjacent to the nucleus, and a single branching mitochondrion was observed (figure 2*a,e*). Mitochondrial cristae of short tubular or vesicle shapes normally contain a filament (figure 2*a,e*, inset), which is characteristic of stramenopiles. The nucleus with the central nucleolus is typically located near the cell centre (figure 2*a,b*) but may move towards the periphery if a large food vacuole is formed (figure 2*c*). Two flagellar kinetosomes lie subapically at the ventral side of the cell. Two flagella emerge from independent flagellar pockets on the ventral side. Cytoskeleton structures consist of microtubular bands and fibres derived from kinetosomes (figures 1*a* and 2*d,f* ).

**Figure 4.**
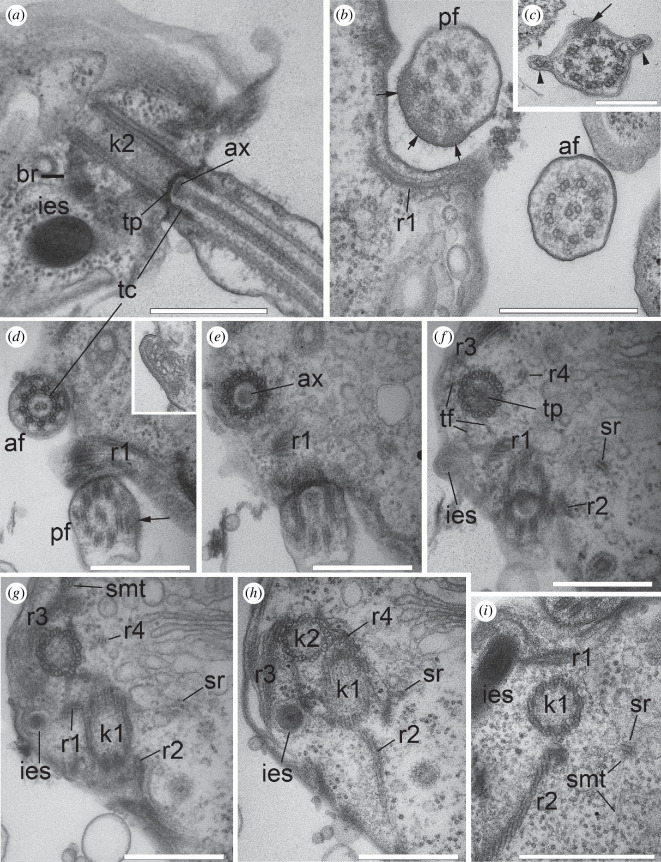
Flagellar apparatus structure in the nibblerids (*a*) *Nibbleromonas kosolapovi*, (*b*,*d*–*i*) *N. quarantinus* and (*c*) *N. arcticus*. (*a*) Longitudinal section (LS) of the kinetosome and anterior flagellum. (*b*) Transverse section (TS) of medium part of the anterior and basal part of the posterior flagella showing a classical structure of the axoneme (9+2). The arrows point the fibrillary matrix in the posterior flagellum faced to surface cavity underlined with the microtubules of r1. (*c*) TS of the posterior flagellum with two opposite folds (arrowheads) and the fibrillar matrix (arrow). (*d*–*h*) Series of TSs of transitional zone (*d*–*f*) and kinetosome of anterior flagellum (*g,h*). (*d*) (inset) r1 consists of four microtubules in the distal part. (*i*) TS of k1 with its roots showing secondary microtubules (smt) from the singlet (sr). All images are deployed as follows: the anterior end of the cell is directed upwards, we are looking at the ventral side of the cell from the outside, as evidenced by a cross section of the kinetosome, the triplets of which are tilted counterclockwise (figure 4*f–h*). If triplets are not visible in sections, we are guided by a wide cross-striated fibrillar bridge connecting kinetosomes on the cell right side. Scale bar: (*a*,*b*,*d*–*i*) 0.5 µm; (*c*) 0.2 µm. Abbreviations: af: anterior flagellum; ax: axosome of flagellum; br: right cross-striated fibrillary bridge between kinetosomes; ies: interflagellar extrusomes; k1: kinetosome of posterior flagellum; k2: kinetosome of anterior flagellum; pf: posterior flagellum; r1–r4: roots of flagella; smt: secondary microtubules; sr: singlet root; tc: transitional cylinder; tf: transition fibrils; tp: transversal plate.

### 3.3. Extrusomes

**Figure 3.**
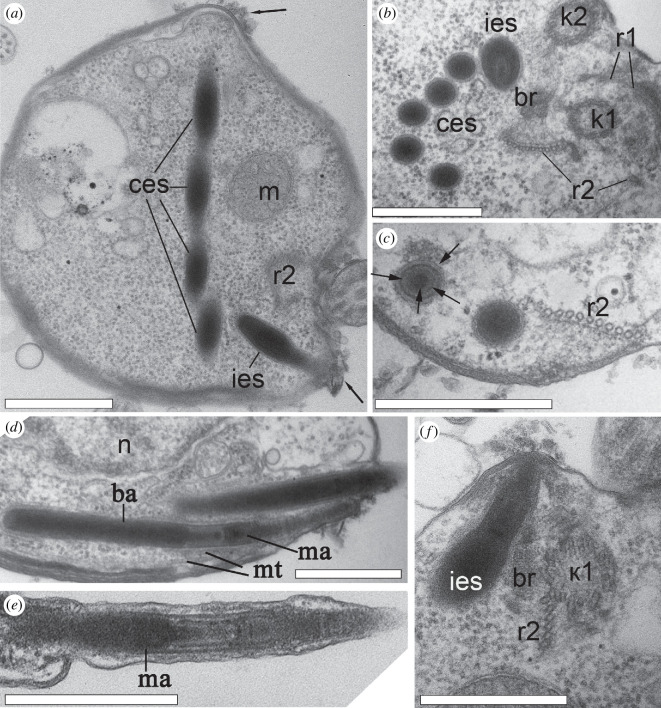
Extrusome structure of the nibblerids *Nibbleromonas quarantinus* (*a–d,f*) and *N. arcticus* (*e*). (*a*) Orthogonal disposition of cytostomal and interflagellar ampulosomes in the cell. The arrow point a small layer of electron-dense pieces, possibly indicating a special adhesive property in this region. (*b*) Full set of five cytostomal ampulosomes at the transverse section (TS) and thick interflagellar ampulosomes at the tangential section in kinetosomes vicinity. (*c*) TS of cytostomal ampulosomes at the level of the ampule head (arrows). (*d*,*e*) Longitudinal sections (LSs) of the cytostomal ampulosomes of the thorn showing their internal structure. (*f*) LS of interflagellar ampulosome lying near k1. Scale bar: 0.5 µm. Abbreviations: ba: basal ampule of the ampulosome; br: right cross-striated fibrillary bridge between kinetosomes; ces: cytostomal extrusomes; ies: interflagellar extrusomes; k1: kinetosome of posterior flagellum; k2: kinetosome of anterior flagellum; m: mitochondrion; ma: middle ampule of the ampulosome; mt: microtubules; n: nucleus; r1–r2: roots of flagella.

Nibblerids have two groups of extrusomes that differ in shape and location. One group of normally five needle-like extrusomes are oriented towards the posterior end of the ventral groove, and called here the cytostomal extrusomes (ces). Another type of extrusive organelles is normally singular, but in rare instances two or three are found, is half the length, ampule-shaped, and lay perpendicular to the other extrusomes, ending between the kinetosomes (figures 2*d,f* and 3*a,b,f*). This type is referred here as interkinetosomal extrusomes (ies). Most likely, extrusomes can move inside the cell due to cytoplasmic microtubules (figure 3*d*). One or two cytostomal extrusomes can extend out of the cell by one micron, forming a thorn (figure 3*e*). The cytostomal and interkinetosomal extrusomes have a similar general morphology of inner structures, but are located in different regions of the cell and differ in shape. The proximal half of the extrusome is filled with electron-dense material forming a basal ampule (figures 1*c* and 3*d*). A thin cylindrical structure connects the pointed tip of the basal ampule to the base of the small middle ampule, which continues into the less osmiophilic and cross-striated needle tapering at the apical point of the extrusome (figures 1*c* and 3*c*,*e*). On the side where the extrusomes make contact with the plasmalemma, a small layer of electron-dense pieces was observed, possibly indicating a special adhesive property in this region (figure 3*a*, arrows). Considering their unique structure, we propose calling them ampulosomes.

### 3.4. Flagellar apparatus

The middle and distal parts of the flagellum have a typical organization: an axoneme 9+2 surrounded by a narrow layer of cytoplasm, which is in turn covered by the plasmalemma. Thin hairs were found on the proximal part of the posterior flagellum inside the flagellar pocket (electronic supplementary material, figure S4e). Closer to the base, the posterior flagellum forms two opposite lateral vanes (folds) shown by the arrowheads, and dilation on one side of the flagellum, often containing a fibrillar seal (arrow) (figure 4*b,c*).

The transition zones of both flagella are similar. The central pair (cp) of microtubules starts from the axosome (ax), which is located on the concave transition plate (figure 4*a,d*–*f*). The base of cp is surrounded by a transitional cylinder (tc). The distal ends of kinetosomes are connected to the plasmalemma by transition fibrils (tf).

To clearly describe the relative positions of each root and their derivatives, all images of the flagellar apparatus were oriented as follows: the anterior end of the cell is directed upwards, and the observer looks at the ventral side of the cell from the outside. This approach is taken because the triplets of kinetosomes are rotated counterclockwise (figure 4*f–h*); therefore, we look at the kinetosome from the flagellum tip to the base. In this case, a broad cross-striated croissant-like fibrillar bridge (br) connects the kinetosomes to each other from their right side (figure 2*a*, inset). If the triplets are not visible in the images, we use this bridge for correct cell orientation. The angle between the kinetosomes ranged from 0° to 60° (figures 1*a,b*, 2*c* and 4*h*).

According to the broadly accepted numbering of kinetosomes and their roots [2], the kinetosome of the anterior flagellum is k2, and the kinetosome of the posterior flagellum is k1.

K1 produces two microtubular roots: root 1 (r1) and root 2 (r2). R1 begins from the point where k1 is connected to the bridge as two microtubules (figures 1*a,b* and 4*g*; electronic supplementary material, figure S2, S4*c*) and continues between the kinetosomes inside the adjacent wall of the flagellar pockets, increasing to four microtubules (figures 1*a*, 2*f*, *4d–f,g,i* and 4*d*, inset). R1 reinforces the left side of the ventral groove (‘left jaw’) (figures 1*a,b*, 2*f*; electronic supplementary material, figure S4*b−e*). The dense fibril originates from the br and is connected to r1 along its entire visible length (figure 1*a*; electronic supplementary material, figure S1*e,f*).

R2 is the main feeding root, which originates from the posterior surface of k1. It consists of three microtubules at its origin and is associated with the fibril plate at the bridge (figures 1*a* and 3*b*; electronic supplementary material, figure S3).

R2 lines the right side of the cytostome (the right ‘jaw’) along the ventral surface of the cell towards the distal end, and the thorn with needle-like ampulosomes (figures 1*a,b* and 2*d,f*; electronic supplementary material, figure S1,4*e–h*). The number of microtubules increases to 12. The plate at the base of r2 is connected to the bridge by a network of thin filaments, which extend along this r2 branch to the distal end. The filaments connect with the plasmalemma at the posterior-ventral end of the cell (figure 3*c*). The long cytostomal fibre originates from the bridge and extends from the distal end of k2 along the entire length of r2 (figures 1*a* and 2*d*; electronic supplementary material, figure S4*e*).

The singlet root (sr) passing from the left side of k1 produces few secondary microtubules underlying the cytostome (figures 1*a,b* and figure 4*f*-*i*; electronic supplementary material, figures S1*a,b* and S3*b–g*). The arrangement of r1 and sr in reinforcing the left ‘jaw’ involves each root passing along different sides of the adjacent pocket wall (figure 1*a,b*; electronic supplementary material, figures S1−S4).

K2 produces root 3 (r3) and root 4 (r4) microtubular roots. R3 originates from the right side of k2, gives a short branch that links to the bridge, and passes anterior-ventral as a band of three microtubules, which produce many secondary microtubules under the dorsal cell surface (figure 1*a*, 2*f* and 4*g,h*, electronic supplementary material, figures S1*a–e*, S2*a–f*, S3*a* and S4*a*). R4 is a singlet root that originates from the left side of k2. It is associated with a thin fibril and lengthens parallel to r3 toward the apical end of the cell (figure 4*f-h*; electronic supplementary material S1*a–d* and S2*a–f*).

### 3.5. External morphology and behaviour of *Nibbleromnas piranha* sp. nov.

**Figure 5.**
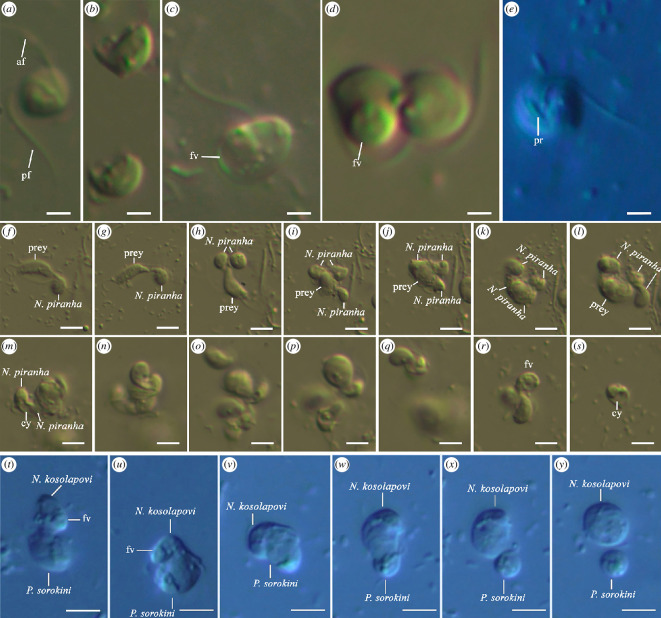
Light microscopy of live cells of (*a*–*s*) *N. piranha* sp. nov. and (*t*–*y*) *N. kosolapovi*. (*a*) General view of the cell. (*b*) Starving cells with thorns. (*c*) Well-fed cell with large food vacuole. (*d*) Dividing cell of *N. piranha* sp. nov. (*e*) The cell with protrusion between flagellar pockets. (*f–l*) An aggressive feeding of *N. piranha* sp. nov. on *P. sorokini* (see §3). (*m*–*s*) Competing feeding behaviour resulting in cannibalism. (*f–l*) Joint feeding of *N. piranha* sp. nov., then (*m–r*) cannibalism occurs. (*s*) The predator with open cytostome. (*t*–*y*) Typical nibbling of *Nibbleromonas* spp. (*N. kosolapovi*; images were obtained from electronic supplementary material, video material) [6]. Scale bars: (*a*–*e*) 2 µm; (*f*–*y*) 5 µm. Abbreviations: af: anterior flagellum; cy: cytostomal ventral groove; fv: food vacuole; pf: posterior flagellum; pr: protrusion; prey: *P. sorokini*.

**Figure 6.**
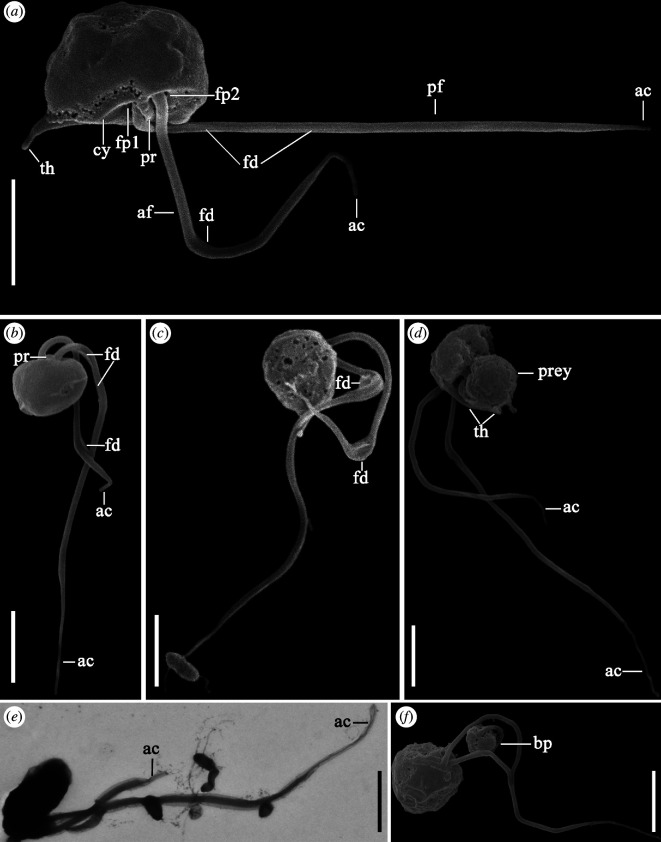
External morphology of nibblerids. Scanning electron microscopy: (*a*–*c*) *N. piranha* sp. nov., (*d*,*f*) *N. kosolapovi*. Transmission electron microscopy: (*e*) *N. piranha* sp. nov. Scale bar: 2 µm. Abbreviations: ac: acroneme; af: anterior flagellum; bp: the bitten prey (*P. sorokini*); cy: cytostomal ventral groove; fd: fold; fp1: flagellar pocket of posterior flagellum; fp2: flagellar pocket of anterior flagellum; pf: posterior flagellum; prey: *P. sorokini*, th: thorn.

The predatory eukaryovorous flagellates *Nibbleromonas piranha* sp. nov. have crescent-shaped cells (figures 5*b* and 6*a*). The cell length is 3.2–5.6 µm, and the cell width is 2.7–4.9 µm. Two heterokont acronematic flagella emerge from independent flagellar pockets, which are separated by a cytoplasmic protrusion (figures 5*e* and 6*a,b*). The ventral feeding groove is located distal to the flagellar pocket of the posterior flagellum (figure 6*a*). There were no mastigonemes on the surface of the flagella (figure 6*a–c,e*). The posterior flagellum is approximately three times longer than the cell body and one and a half times longer than the anterior flagellum (figure 6*a–c*). Both flagella have two keel-like folds that extend from the proximal quarter of the posterior flagellum and distal third of the anterior flagellum. Folds are located ventrally and dorsally relative to the cell body (figure 6*a–c*). There was a clear dimorphism of well-fed and starving cells (figure 5*b,c*). The size of the cell can increase up to twofold after feeding. Starving cells impulsively spin around their axis, staying in one place or floating with the apical part forward, changing the direction of movement situationally. Well-fed cells lie at the bottom of a Petri dish or move quickly but more smoothly and do not abruptly change the direction of movement. Small starving cells are not able to consume an entire *P. sorokini* cell completely, so they bite off most of the cell of the prey (video 1; figure 6*f*), like other species of *Nibbleromonas* (figure 5*t–y*) or, sometimes, feed jointly (figure 5*f–l*). *Nibbleromonas* attacks *P. sorokini* cell by attaching to it with a distal thorn and then apparently immobilizing the prey using ampulosomes located inside the thorn. The prey becomes rounded and stops moving, at which time the ventral part of the predator cell comes into closer contact with the prey (figures 5*f* and 6*d*). *N. piranha* sp. nov. stretches over the prey and opens the cytostome (figures 5*s* and 6*d*). Both flagella wrap around the prey body, helping to hold and engulf it. Sometimes, one of the flagella is attached to the bottom of the Petri dish (video 2). After this, the size of the *Nibbleromonas* cells increased significantly. These cells continue to feed and can engulf *P. sorokini* cells entirely, forming a large food vacuole (figure 5*c*). Well-fed cells do not have a thorn in the distal part and are pear-shaped (figure 5*c*; videos 1,3). Cysts were not observed in the life cycle. The flagella became shorter and duplicated at the first stage of cell division (figure 5*d*; video 4). Then, a pair of flagella moves to the posterior end of the cell, and cytokinesis occurs. At the end of cytokinesis, the two daughter cells are located upside down to each other. One daughter cell inherits a large food vacuole (figure 5*d*), and another, a slightly smaller cell inherits a thorn (video 4).

Joint feeding occurs when predators attach to an immobilized prey that is already being eaten by another cell of *Nibbleromonas piranha* sp. nov. In this case, predators consume the prey from different sides, with one of them engulfing most of prey’s cell (or whole cell), while the others competing for prey and attacking each other with ampulosomes (figure 5*f–l*). When two *N. piranha* sp. nov. cells ate the same prey from different sides, one of them always eventually stopped feeding and slipped away. Approximately, 10 cells were found trying to eat a single *P. sorokini* cell, and some of them demonstrated cannibalism (video 3, figure 5*f–s*). The feeding behaviour of *N. piranha* sp. nov. differs from that of other known *Nibbleromonas* species because nibbling of the prey is less frequent compared with eating whole prey, and competition for the same prey cell with other individuals is more common.

### 3.6. 18S rRNA gene phylogeny

**Figure 7.**
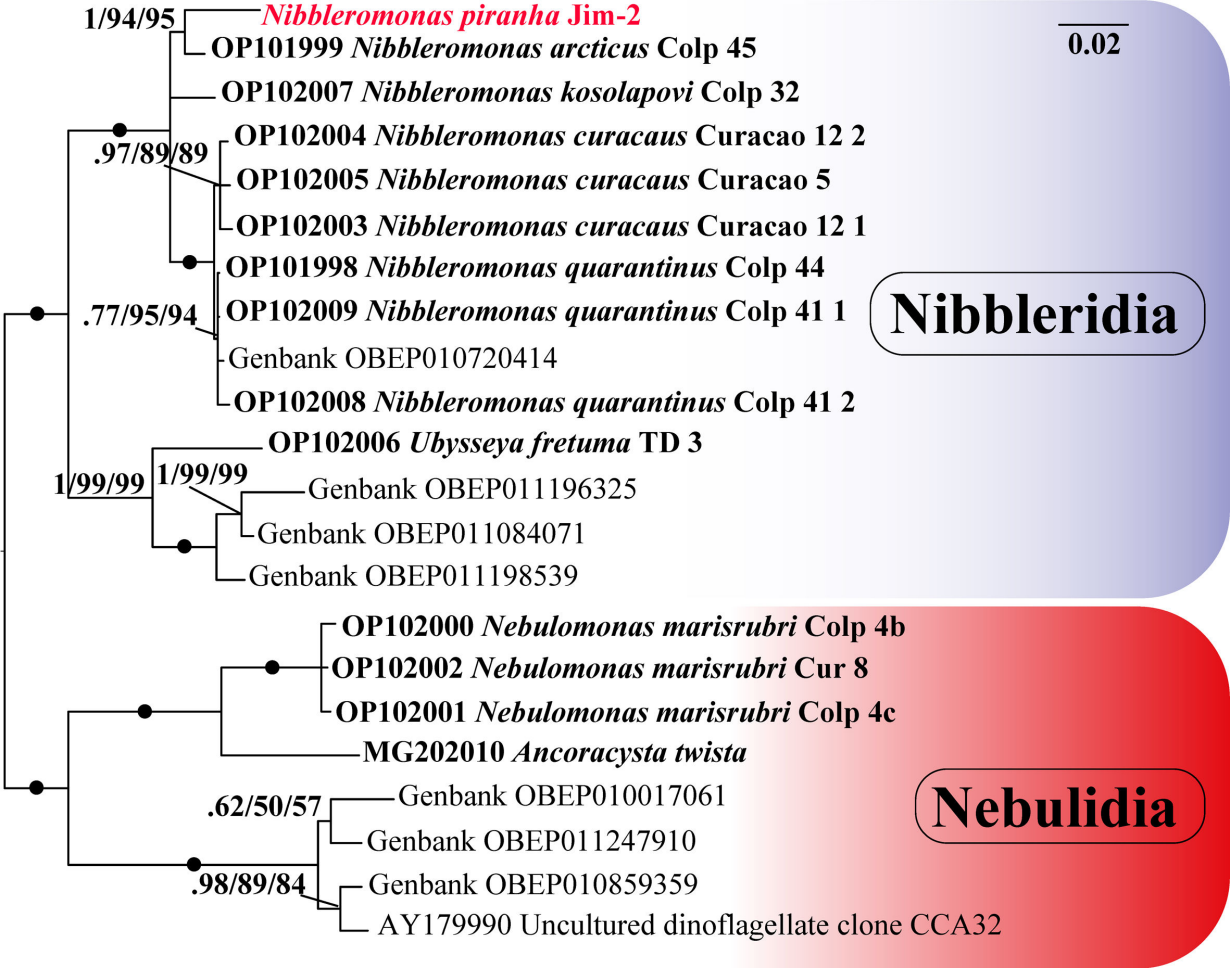
The 18S rRNA phylogeny of Provora. Branch nodes show MrBayes posterior probability/IQ-TREE standard bootstrap/RAxML standard bootstrap support values. The black dots indicate full support (100/1/100). Taxa labels in bold font were previously annotated in GenBank as species.

We carried out phylogenetic analysis with all available provorans 18S rRNA sequences. Provora has been clearly divided into two groups, Nibbleridia and Nebulidia, with full BI and ML support (figure 7). *Ubyssea* and three related environmental sequences form a separate lineage from the genus *Nibbleromonas* with nearly full support (figure 7). *N. piranha* sp. nov. belongs to a clade containing *Nibbleromonas* species and the OBEP010720414 environmental sequence (figure 7). *N. piranha* sp. nov. was most closely related to OP101999 *N. arcticus,* which was fully supported in Bayesian analysis (figure 7) and highly supported in the RAxML and IQ-TREE reconstructions (figure 7).

**Figure 9.**
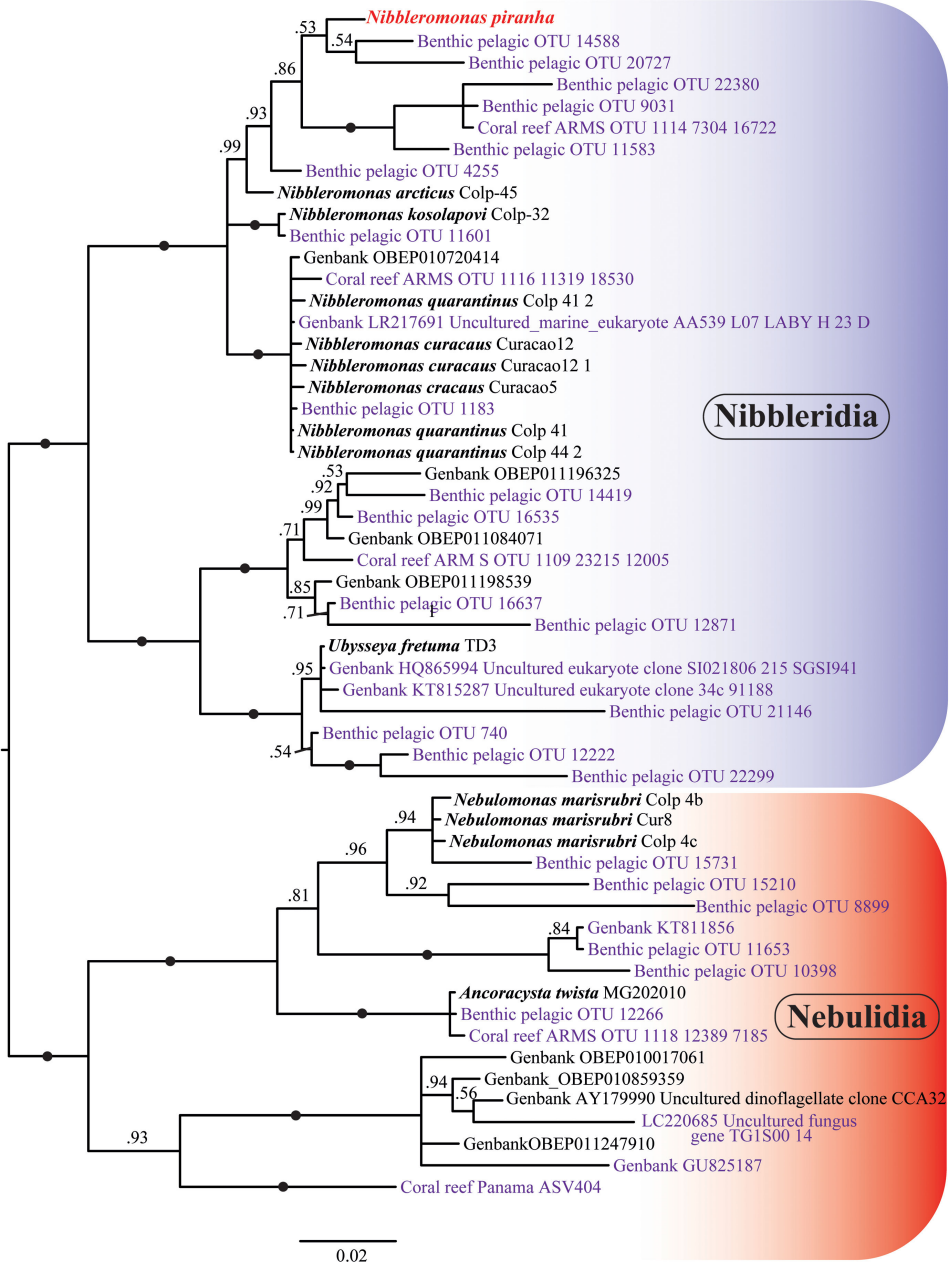
The phylogeny of Provora. Bayesian phylogeny including environmental short-read sequences (≥360 bp) of 18S rRNA. Branch nodes show MrBayes posterior probability support values. The black dots indicate full support (1). The taxa in violet indicate environmental short-read sequences, and the taxa in bold indicate species that were previously annotated in GenBank.

**Figure 8.**
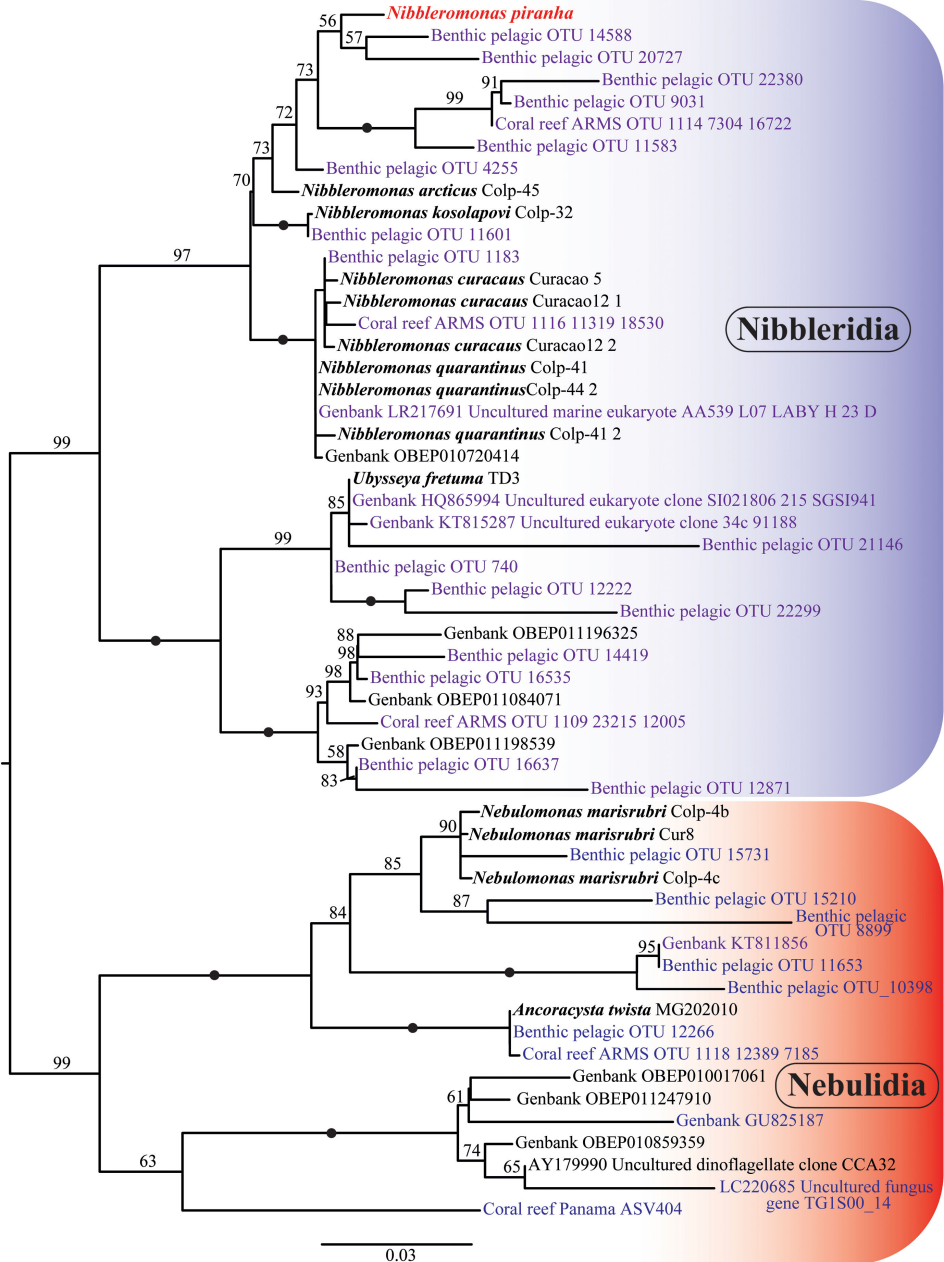
The phylogeny of Provora. IQ-TREE phylogeny including environmental short-read sequences (≥360 bp) of 18S rRNA. Branch nodes show IQ Tree standard bootstrap support values. The black dots indicate full support (100). The taxa in violet indicate environmental short-read sequences, and the taxa in bold indicate species that were previously annotated in GenBank.

In addition, we reconstructed an unrooted tree of Provora with short-read environmental 18S rRNA sequences. Even with short reads the high support in the main nodes was maintained, the branching order remained basically unchanged compared with trees based on longer sequences (figures 7–9). *N. kosolapovi*, *N. arcticus*, *N. quarantinus*, *N. curacaus* and *N. piranha* sp. nov. all branched within clades with several marine environmental sequences with high support (figures 8 and 9). The relationships between our newly discovered strain and the uncultured sequences have not yet been fully determined, but we found that they were placed in a separate lineage from *N. arcticus* with high support in Bayesian analysis (0.99) and with moderate support (73%) in the IQ-TREE reconstruction (figures 8 and 9).

The phylogenetic analysis with wide sampling of eukaryotic taxa showed that *N. piranha* sp. nov. was placed within the *Nibbleromonas* clade, with high support. Similarly to the unrooted trees, Provora was divided into two monophyletic groups: Nibbleridia and Nebulidia, albeit with low support (electronic supplementary material, figure S5).

## 4. Discussion

The phylogenetic position of Provora has not yet been determined unambiguously, but phylogenomics has shown it is an ancient lineage and narrowed down the range of possible position in the tree of eukaryotes to a few more likely candidates. Ultrastructural features can help us narrow down their likely evolutionary relationships further, and together with molecular trees these characters are also important for reconstructing how key features of the eukaryotic cell evolved. In particular, the centriole-associated skeleton has often been used to guide systematics [14,15,26], and is also a system central to understanding how eukaryotic cells evolved and diversified. The provorans centriole cytoskeleton has an interesting mix of unique and potentially widespread ancestral features, so we will discuss these features in particular detail, with particular emphasis on two possible phylogenetic placements of Provora on the eukaryotic tree as suggested by phylogenomics: as sister to the TSAR+Haptista group, or alternatively as sister to Hemimastigophora [6].

Having vanes on both flagella like *Nibbleromonas* is rarely found in other eukaryotes. However, the vane of the posterior flagellum is a typical trait for malawimonadids, discobids and metamonads [13,27–29]. Furthermore, it is worth mentioning that some bicosoecids exhibit dorsal swellings on both flagella [30], but without fibres inside. Interestingly, the two vanes on the posterior flagellum (PF) have been argued to be an ancestral state in eukaryotes [27,29,31], but in no other group have such vanes been described on the anterior flagellum (AF). We believe that the malawimonads *Imasa* and *Gefionella* may also have vanes on their anterior flagella, which was not noticed by Heiss *et al*. [32, fig. 1f; 31, fig. 2D]. If our assumptions are correct, then double vanes on both flagella might be a more ancient character than presently appreciated. In *Nibbleromonas*, the ventral vane originates on the proximal side of the flagellum, while the dorsal vane is positioned slightly more distally to it. These vanes pass together to form the opposite vanes of the PF. Subsequently, electron-dense material appears in a dilatation on the other side. The same pattern was identified, but with a modified or absent dilation for malawimonads, metamonads and some stramenopiles [13,32–35]. The proximal part of the ventral posterior vane of *Malawimonas* is remarkably ultrastructurally similar to that of *Nibbleromonas* (electronic supplementary material, figure S3*h*,*i*) [6, fig. 1t; 27, fig. 6]. *Nibbleromonas* vanes exhibit an obvious size reduction that would be useful for active swimmers and hunting predators, such as nibblerids, as well as nebulids, colponemids and stramenopiles. However, unlike most of them, nibblers often change their direction of movement and rapidly rotate around their own axis, which is accompanied by a dynamic beating of their flagella. It is known that small dorsal posterior vanes are found in *Ubyssesa* and nebulid species, but they do not have any vanes on their AF [6,36]. If these are due to reduction of vanes on the AF and the ventral vanes on the PF, it would suggest that *Nibbleromonas* uniquely retains these ancestral features in flagellar architecture. Different paraxial formations have also been identified in many other lineages of protists, including Metamonada, Discoba, Alveolata, Stramenopile, Malawimonadidae and Provora, but we cannot exclude the convergence of these features.

The inner structure of the ampulosomes in *Nibbleromonas* are similar to the extrusive organelles in *Hemimastix amphikineta* and *Stereonema geiseri* [37,38]. Both organelles have an electron dense matrix in their proximal part and have a sharpened distal end, which contacts the plasmalemma, but the ampulosomes of *Nibbleromonas* contain three (proximal, middle and distal) ampules. The extremely complex and similar structures of these types of extrusomes are unique to Provora and Hemimastigophora. This complexity argues against independent origins, especially when at least some trees place the two groups as sisters [6, extended data fig. 1b]. At the same time, a general view of the cell, mitochondrial and flagellar apparatus structures in these groups are quite different and does not provide many insights into their close relationships. Moreover, complex extrusomes in other groups like dinoflagellates have been argued to have been lost based on their phylogenetic distribution within the group [39], so the extrusomes in Provora and Hemimastigophora may similarly be considered to be homologous without arguing the two groups are sisters.

### 4.1. The ventral feeding groove of nibblerids reinforced by r2 and r1

The r2 is similar to that of malawimonads, metamonads, jakobids [13,28,33] and some early divergent stramenopiles [40,41], if we assume that Bicosoecida is one of the deep branches of the Stramenopile tree [42–44]. The wide part of r2 has a fibril plate with a fibrous connective with a fibril bridge, which appears similar to the architecture of the I-fibre and fibrous material of malawimonads [32, fig. 3d,g], metamonads [34, fig. 28; 33, fig. 4a,b; 29, fig. 7] and jacobids [28, fig. 5]. These structures could be homologues to the electron-dense material of R2 [41, fig. 5a] and filamentous connective of R2 [41, fig. 4d] seen in some phagotrophic stramenopiles, while the deepest branching stramenopile do not have any fibrils [40]; however, figure 4 of [41] suggests the I-fibre may lay near R2. *Platisulcus tardus* has a ventral groove and four main flagellar roots +S tubule. The R2 of *P. tardus* is split into inner and outer parts. The *Nibbleromonas* r2 also has a secondary microtubular band, which in turn creates another secondary band on the left side of the cytostome, which is only visible during the feeding process. Notably, sr is an atypical case, as it provides secondary microtubules and could be the right side of r2 or its functional equivalent. Unlike that of nibblerids, the R2 of *P. tardus* dissipates towards the distal end of the cell. By contrast, in *Nibbleromonas*, the number of microtubules increases from the apical end to the distal end. Some bicosoecid flagellates likely have wide strong feeding roots (arranged in a ventral groove), but they are associated with the younger kinetosome of the anterior flagellum (k2) or its fibres [30]. However, the filamentous connectives of fp (the fibrillar bridge between kinetosomes) associated with r3 of *Regin rotiferus* look similar to the fibrillar plate and r2 of *Nibbleromonas* [30, fig. 3]. Additionally, the I-fibre can be found in deep-branching alveolates [15; 16, fig. 5B].

The r1 consists of two microtubules at its proximal end and is most likely homologous to r1 of chrysophytes and related ochrophytes, which also have two microtubules [45]. The dense fibril of its location and architecture could be equivalent to a C-fibre, which has been observed in many branches of eukaryotes [1].

The r3 shares common features with that of *Malawimonas* [27], *Carpediemonas* [33] and *Platysulcus* [40], but their r3 does not split into two parts. The only description of a similar structure is from *Giraudyopsis stellifer* [45,46], which was marked as a bypassing rootlet. This is interesting, coupled with assumptions about the appearance of the bypassing band (BB) [15].

It has been proposed that the r4 could potentially arise independently in different lineages [15], similar to the chiral root of the r2, sr or as a modification of the anterior root (AR) of excavates. It could be related to its reduction or, possibly, the transformation of this root into a r4 singlet to provide more successful support for AF. This is also important for Provora, which have fast and mobile movement of cells. However, *Platisulcus* has both singlet roots R4 and S tubule [40], unlike *Rictus,* which has only the SR [41, fig. 6].

Overall, the ultrastructure of vanes, the presence of alveoles under the plasmalemma, and the main architecture of the flagellar apparatus suggest that the possible ancestor of such superclusters as TSAR+Haptista and Archaeplastida+Cryptista was an alveolate-like phagotrophic flagellate with a ventral groove and flagellar vanes on both flagella [15,47].

### 4.2. External morphology and behavioural features of *N. piranha* sp. nov.

The typical characteristic of the genus *Nibbleromonas* is the presence of a thorn on the ventro-caudal side of the cell [6]. This structure contains several ampulosomes that help to immobilize and capture prey. All known species of *Nibbleromonas* exhibit a classic predator‒prey feeding strategy. Additionally, *N. piranha* sp. nov. demonstrates unique behaviour and attacks individuals of its own species, resulting in cannibalism.

Biting off a larger portion of the prey cell is a distinguishing feature of *N. kosolapovi*, *N. arcticus* and *N. quarantinus*. As a result, the unconsumed part of the prey separated into a small vesicle, which was seemingly consumed by bacteria rather than by another predator. It seems that *N. piranha* sp. nov. rarely employs this feeding strategy. This species consumes *P. sorokini* completely, or if the predator is starving and much smaller than the prey, several cells feed on a single prey jointly. Joint feeding is also observed in some other flagellates, such as opistokont protists and colpodellids, but this behaviour has never been observed to result in cannibalism or attacks on one another [3,4]. This appears to occur by mistake, leading to cannibalism, a behaviour observed in some other protists as well [48–51].

*N. piranha* sp. nov. demonstrates a higher frequency of competing feeding than other species of *Nibbleromonas*. The reason for this and mechanism by which *Nibbleromonas* cells are attracted to prey are currently unknown. However, it is possible that *Nibbleromonas* predators, such as *N. piranha* sp. nov., may excrete signalling molecules during feeding. These molecules could potentially be up-regulated in response to feeding and encoded by genes involved in cell signalling, similar to those in predatory opisthokonts [52] or the extraction of gamons in ciliates [53]. When a predator is already feeding and another *N. piranha* sp. nov. attacks the same prey, it can become attached to the thorn to the first predator, leading to cannibalistic feeding. These mistakes are deleterious evolutionarily, but perhaps the benefit of competitive feeding over nibbling balances favourably against this disadvantage, since it results in the complete consumption of large prey. Further research is needed to explore and understand the specific mechanisms of *Nibbleromonas* cell attraction.

## 5. Conclusion

The relationship between Provora and other supergroups requires more data and analysis, but will likely be clarified through multigene molecular phylogenetics once more diversity of this supergroup at the level of different genera and families accumulate. The general plan of the external morphology and cytoskeletal system of *Nibbleromonas* spp. appears to shed light on the ancestral state of several major lineages of eukaryotes, but careful analysis of the ultrastructure also suggests that Provora share particular morphologically similarities to the TSAR+Haptista grouping. The typical mode of feeding in nibblerids results from the coordinated acts of several microtubular roots (r1, sr and r2), which reinforce the cytostome groove and undergo dynamic polymerization–depolymerization during the process of ingesting and biting off larger prey. The wider band (r2) forms the right ‘jaw’ of the cytostome, while the singlet (sr) and r1 work as the left ‘jaw’. A new type of extrusive organelle, the ampulosome, consists of three ampule-like structures with an electron-dense matrix and appears to be homologous to Hemimastigophora extrusomes. The frequent collective feeding observed is likely related to the biochemical nature of intercellular signalling. Considering the global distribution of provorans species, it is essential to identify new strains and conduct a more comprehensive morphological, phylogenomic and transcriptomic analysis to fully understand this mysterious predatory group and its role in aquatic ecosystems.

## 6. Taxonomic summary

Assignment. Eukaryota; Provora; Nibbleridia; Nibbleridea; Nibbleridida; Nibblerididae; *Nibbleromonas*

*Nibbleromonas piranha* sp. nov. Belyaev, Tikhonenkov et Karpov

Diagnosis. Cells are 3.2−5.6 µm long and 2.7−4.9 µm wide. Starving cells have thorn on the distal end, while well-fed cells are typically pear-shaped and without thorn. Heterokont acronematic flagella 4−7 µm (anterior) and 7−10 µm (posterior) in length possess two opposite folds (vanes). Cysts were not observed. The organism exhibits aggressive competitive feeding behaviour with cannibalistic attempts.

Type figure. Figure 6*a* illustrates a cell of strain Jim-2.

Type locality. Coastal marine sediments of Sea of Japan, Jeodo island, Republic of Korea.

Etymology. Named after fish from the Serrasalmidae family due to their aggressive behaviour.

Gene sequence. The 18S rRNA gene sequence has the GenBank Accession Number PQ417912

Zoobank Registration. urn:lsid:zoobank.org:act:47C60B3B-D3AE-485C-AA63-1AC848D9F7ED

## Data Availability

All data are available in the main text and on Figshare [54]. Zoobank Registration. urn:lsid:zoobank.org:act:47C60B3B-D3AE-485C-AA63-DNA sequences: GenBank accession number PQ417912. Supplementary material is available online [55].
